# PHB2 promotes SHIP2 ubiquitination via the E3 ligase NEDD4 to regulate AKT signaling in gastric cancer

**DOI:** 10.1186/s13046-023-02937-1

**Published:** 2024-01-11

**Authors:** Liang Xu, Wanying Xiang, Jiezhen Yang, Jing Gao, Xinyue Wang, Li Meng, Kaihong Ye, Xiao Hong Zhao, Xu Dong Zhang, Lei Jin, Yan Ye

**Affiliations:** 1 Department of Immunology, School of Basic Medical Sciences, Anhui Medical University, Hefei, 230032 Anhui China; 2 School of Biomedical Sciences and Pharmacy, The University of Newcastle, Newcastle, NSW 2308 Australia; 3 Department of Pathology, Zhongshan Hospital (Xiamen Branch), Fudan University, Xiamen, 361015 China; 4 Translational Research Institute, Henan Provincial and Zhengzhou City Key Laboratory of Non-Coding RNA and Cancer Metabolism, Henan International Join Laboratory of Non-Coding RNA and Metabolism in Cancer, Henan Provincial People’s Hospital, Academy of Medical Sciences, Zhengzhou University, Zhengzhou, 450053 Henan China; 5 School of Medicine and Public Health, The University of Newcastle, Newcastle, NSW 2308 Australia

**Keywords:** PHB2, SHIP2, NEDD4, Ubiquitination, Gastric cancer

## Abstract

**Background:**

Prohibitin 2 (PHB2) exhibits opposite functions of promoting or inhibiting tumour across various cancer types. In this study, we aim to investigate its functions and underlying mechanisms in the context of gastric cancer (GC).

**Methods:**

PHB2 protein expression levels in GC and normal tissues were examined using western blot and immunohistochemistry. PHB2 expression level associations with patient outcomes were examined through Kaplan–Meier plotter analysis utilizing GEO datasets (GSE14210 and GSE29272). The biological role of PHB2 and its subsequent regulatory mechanisms were elucidated in vitro and in vivo. GC cell viability and proliferation were assessed using MTT cell viability analysis, clonogenic assays, and BrdU incorporation assays, while the growth of GC xenografted tumours was measured via IHC staining of Ki67. The interaction among PHB2 and SHIP2, as well as between SHIP2 and NEDD4, was identified through co-immunoprecipitation, GST pull-down assays, and deletion-mapping experiments. SHIP2 ubiquitination and degradation were assessed using cycloheximide treatment, plasmid transfection and co-immunoprecipitation, followed by western blot analysis.

**Results:**

Our analysis revealed a substantial increase in PHB2 expression in GC tissues compared to adjacent normal tissues. Notably, higher PHB2 levels correlated with poorer patient outcomes, suggesting its clinical relevance. Functionally, silencing PHB2 in GC cells significantly reduced cell proliferation and retarded GC tumour growth, whereas overexpression of PHB2 further enhanced GC cell proliferation. Mechanistically, PHB2 physically interacted with Src homology 2-containing inositol 5-phosphatase 2 (SHIP2) in the cytoplasm of GC cells, thus leading to SHIP2 degradation via its novel E3 ligase NEDD4. It subsequently activated the PI3K/Akt signaling pathway and thus promoted GC cell proliferation.

**Conclusions:**

Our findings highlight the importance of PHB2 upregulation in driving GC progression and its association with adverse patient outcomes. Understanding the functional impact of PHB2 on GC growth contributes valuable insights into the molecular underpinnings of GC and may pave the way for the development of targeted therapies to improve patient outcomes.

**Supplementary Information:**

The online version contains supplementary material available at 10.1186/s13046-023-02937-1.

## Introduction

Prohibitin 2 (PHB2, also known as Prohibitone, REA or BAP37) along with its homolog Prohibitin (PHB or PHB1), belong to the PHB domain family. These proteins are highly conserved in eukaryotic cells and exhibit ubiquitous expression [1, 2]. PHB proteins comprise an N-terminal transmembrane domain, an evolutionarily conserved PHB domain, and a C-terminal coiled-coil domain, which facilitates their interaction in the inner mitochondrial membrane, thereby stabilizing mitochondria [1]. Beyond their localization in the inner mitochondrial membrane, PHBs are also found in the cytoplasm and nucleus, where they regulate vital cellular processes, including cell metabolism, inflammation, migration, apoptosis, and survival [3–8].

Accumulating evidence has revealed the significant involvement of PHB2 in various biological processes related to tumorigenesis [9]. Moreover, it is commonly expressed at high levels and played oncogenic roles in multiple cancers, such as non-small cell lung cancer (NSCLC), esophageal squamous cell carcinoma (ESCC), hepatocellular carcinoma, and prostate cancer, compared to normal tissues [10–13]. Additionally, numerous studies have observed a negative correlation between PHB2 expression and the prognosis of tumour patients in various cancer types [10, 11, 14]. PHB2 modulates various signaling pathways through its interactions with numerous functional proteins, thereby impacting the survival, proliferation, and migration of cancer cells [9, 15, 16]. For instance, in NSCLC, PHB2 interacts with receptor for activated C kinase 1 (RACK1), stabilizing it through post-translational modification. This interaction activates downstream tumour-promoting effectors, such as Akt and FAK [10]. Furthermore, PHB2 accelerated colorectal cancer cell proliferation and promoted tumorigenesis via NDUFS1-mediated oxidative phosphorylation [4]. However, PHB2 was discovered as a tumour suppressor in other cancer types. For instance, PHB2 acts as a tumour suppressor in breast cancer by interacting with estrogen receptor-alpha (ERα) in the nucleus, where it functions as a corepressor, inhibiting ERα-mediated transcription and proliferation [17]. Given the intricate functional roles of PHB2 observed across various cancer types, further investigation is warranted to fully elucidate its specific role in each type of cancer. In this study, our aim is to explore the functional role of PHB2 and its underlying mechanisms in gastric cancer (GC).

Src homology 2-containing inositol 5-phosphatase 2 (SHIP2) belongs to the phosphoinositide 5-phosphatase family and plays a critical role in various cellular pathways by hydrolyzing PtdIns(3,4,5)P3 to produce PtdIns(3,4)P2, a negative regulator of phosphoinositide 3-kinase (PI3K) and insulin signaling [18]. SHIP2 comprises an N-terminal SH2 domain, a central catalytic 5-phosphatase domain, a C-terminal proline-rich domain (PRD), and a sterile alpha motif (SAM) [19]. As a scaffolding protein, SHIP2 interacts with different proteins and contributes to various aspects of cellular biological functions [20]. An increasing body of evidence has implicated SHIP2 in the development of numerous cancer types. For instance, suppression of SHIP2 promotes cell proliferation and metastasis by interacting with c-cbl and influencing epidermal growth factor receptor (EGFR) turnover, thereby enhancing EGF-induced Akt activation in breast cancer [21–23]. In hepatocellular carcinoma cells, SHIP2 accumulates polyubiquitination due to its interaction with S-phase kinase-associated protein 2 (SKP2), a component of the E3 ubiquitin ligase complex. Moreover, the downregulation of SHIP2 by Hepatitis B Virus X promotes cell migration and induces resistance to 5-Fluorouracil (5-FU) in hepatocellular carcinoma through SKP2 [24]. In GC cells, the IQ motif containing the GTPase-activating protein 2 (IQGAP2) functions as a binding partner for SHIP2. This interaction enhances SHIP2 phosphatase activity, resulting in the inhibition of GC cell metastasis through suppression of the PI3K/Akt signaling pathway [25, 26].

Given the frequent upregulation of PHB2 in GC, our study aimed to elucidate the involvement and underlying mechanisms of PHB2 in GC. To explore this, we utilized immunoprecipitation followed by mass spectrometry analysis, uncovering SHIP2 as a novel protein binding partner of PHB2. Subsequent validation of this physical interaction in the cytosol of GC cells demonstrated that PHB2 specifically interacts with the SAM domain of SHIP2. Furthermore, PHB2 regulates SHIP2 expression by facilitating its binding to the E3 ligase NEDD4, leading to the degradation of SHIP2 protein primarily through lys-48-linked polyubiquitination. Consequently, this degradation initiates the activation of Akt, thereby promoting the proliferation of GC cells. Notably, the expression of SHIP2 showed a negative correlation, whereas p-Akt expression exhibited a positive correlation with PHB2 expression in GC patient samples. These findings emphasize the intricate regulatory mechanisms underlying the interaction between PHB2 and SHIP2, highlighting the crucial role of this pathway in the progression of GC.

## Materials and methods

### Cell culture and human tissue

The human GC cell lines AGS, HGC-27, MKN-28, MKN-45, the normal gastric mucosal epithelial cell line GES-1, and the human embryonic kidney cell line HEK293T were obtained from the Institute of Biochemistry and Cell Biology, Shanghai Institutes for Biological Sciences, Chinese Academy of Sciences. AGS cells were cultured in F-12 HAM’S (HyClone, USA), HGC-27, MKN-28, and MKN-45 cells were cultured in RPMI-1640 (HyClone, USA), and GES-1 and HEK293T cells were cultured in DMEM (HyClone, USA), supplemented with 10% FBS (Gibco, USA), 1% Penicillin–Streptomycin Solution at 37℃, 5% CO_2_. A total of 49 pairs of GC tissues and matched adjacent non-tumour tissues were collected from patients at the Department of Gastrointestinal Surgery, The First Affiliated Hospital of Anhui Medical University (Hefei, China).

### Tissue Microarray (TMA) Analysis

The PHB2 protein expression in GC tissues was examined using the TMA XT17-035 (Shanghai Outdo Biotech Company, China) including 90 cases of GC with TNM classification and pathology grades of the tumours. Images were captured using Aperio ImageScope and Immune Reaction Scores (IRS) were obtained from two independent pathologists.

### Antibodies and reagents

Antibodies against PHB2 (Cat#14085S), PHB1 (Cat#2426S), SHIP2 (Cat#2839S), Akt (Cat#9272S), pAkt-Ser473 (Cat#4060S), pAkt-Thr308 (Cat#13038S), p21 (Cat#2947S), p27 (Cat#3686S), Histone H3 (Cat#9715S), K63 (Cat#5621S), K48 (Cat#8081S), and Ki67 (Cat#34330SF) were purchased from Cell Signaling Technology (Beverly, MA, USA), antibodies against tGFP (Cat#TA150041) was purchased from OriGene (Rockville, MD, USA), antibodies against Flag (Cat#66008-3-Ig, Cat#20543-1-AP), HA (Cat#51064-2-AP, Cat#66006-2-Ig), His (Cat#66005-1-Ig), GST (Cat#66001-2-Ig), Ubiquitin (Cat#10201-2-AP), β-actin (Cat#66009-1-Ig), NEDD4 (Cat#21698-1-AP), SIAH2 (Cat#12651-1-AP), WWP2 (Cat#67274-1-Ig), Cul4A (Cat#14851-1-AP) and MUL1 (Cat#16133-1-AP) were purchased from Proteintech (Wuhan, China). MG132 (Cat#S1748) was purchased from Beyotime Biotechnology (Shanghai, China). Cycloheximide (CHX) solution was purchased from Sigma-Aldrich (St. Louis, MO, USA).

### Plasmids and transfection

To generate the plasmids, the specific primers were used to synthesize the PHB2, PHB1, and SHIP2 sequences, which were subsequently subcloned into the p3 × FLAG-Myc-CMV24, pCMV-HA, and PCMV6-AC-GFP vectors, respectively. The HA-NEDD4 plasmid (Cat #27002) was obtained from Addgene. The details of specific primers used for synthesis are provided in Supplementary Table 1. Cells were cultured in Opti-MEM medium (Invitrogen) and transfected with 2–4 µg of plasmid or the empty vector (used as a control) using Lipofectamine 2000 reagent (Invitrogen) following the manufacturer’s instructions.

### Total RNA isolation and Quantitative Real-Time PCR (qRT-PCR)

Total RNA was isolated from cultured cells with TRIzol Reagent (Invitrogen) according to the manufacturer’s protocol. Specific primers and Power SYBR Green PCR Master Mix (Applied Biosystems) were used to amplify cDNA after reverse transcription of RNA samples by 5 × HiScript®II qRT SuperMix (Vazyme Biotech, China). RNA expression levels were normalized to GAPDH expression. The specific primers for SHIP2 were as follows: 5’-ATGCCTCAGATGGGGAGGAT -3’(sense), 5’- CATTGGGAGCACTCTCAGCA -3’(antisense); for PHB2, 5’- CAGAGCTGAGCTTTAGCCGA -3’ (sense), 5’- CTGCACAATTTTCTGCCGCT -3’ (antisense); for NEDD4, 5’- CTGGAAGCGTTCGGAAATGG -3’ (sense), 5’- CACGTAAGGATCACTAGCTCCC -3’ (antisense); for GAPDH, 5’- GGACCTGACCTGCCGTCTAG -3’ (sense), 5’- GTAGCCCAGGATGCCCTTGA -3’ (antisense).

### Western blot

Whole-cell lysates were obtained using RIPA buffer (Beyotime Biotechnology, Shanghai, China) and protein samples were separated with SDS–polyacrylamide gel electrophoresis. After transferring them to Nitrocellulose Membranes (Millipore, Burlington, MA, USA), membranes were blocked with 5% skim milk at room temperature for 2 h and incubated them with primary and secondary antibodies. Blots were visualized with a Tanon 4500SF image system (Tanon, Shanghai, China), using β-actin as a control.

### RNA interference

Small interfering RNA (siRNA) and short hairpin RNA (shRNA) were purchased from GenePharma (Shanghai, China) and GeneChem (Shanghai, China), respectively. The sequences were listed in Supplementary Table 2. For NEDD4 siRNA, the specific siRNA sequences were transfected according to the manufacturer’s protocol. For lentivirus-mediated shRNAs against PHB1, PHB2 and SHIP2, specific shRNA sequences were cloned into the lentiviral vector GV248, respectively. The lentivirus was produced and introduced into cells according to the manufacturer’s protocol.

### Immunohistochemistry (IHC)

IHC staining and quantitation were performed as described previously [27]. Briefly, antigen retrieval was performed in a pressure cooker for 30 s at 125℃. Antibody detection was performed using the Dako Envision HRP Detection system/DAB as per the manufacturer’s instructions. Slides were counterstained with Azure B to differentiate the melanin from the brown DAB immunolabeling.

### Immunoprecipitation

Immunoprecipitation was carried out as described previously [26]. Briefly, cell extracts were mixed and precipitated with antibody and protein A/G agarose beads by incubation at 4℃. The bound proteins were removed by boiling in SDS buffer and resolved in SDS-PAGE gels for immunoblotting analysis.

### Mass spectrometry

The immunoprecipitation sample (50 μg) was mixed with UA buffer (8 M Urea, 150 mM Tris–HCl, pH 8.0), followed by the addition of DTT and iodoacetamide to reduce and block cysteine residues, respectively. Next, 12.5 ng/μl trypsin in 25 mM NH4HCO3 was added, and the mixtures were incubated at 37℃ for 16–18 h. Each fraction was separated using the nanoflow HPLC liquid chromatography system (Easy nLC). The chromatographic column was equilibrated with 95% buffer A (0.1% formic acid), and the samples were injected onto the loading column (Thermo Scientific Acclaim PepMap100, 100 μm*2 cm, nano Viper C18) via an autosampler. Separation was achieved using a linear gradient of buffer B (84% acetonitrile and 0.1% formic acid) at a flow rate of 300 nl/min controlled by IntelliFlow technology. After chromatographic separation, the samples were subjected to mass spectrometry analysis using the Q-Exactive mass spectrometer. The analysis duration was set at 120 min. The detection mode was positive ion mode, and the mass range for the precursor ion scan was 300—1800 m/z. The primary mass spectrometry resolution was set at 70,000 at 100 m/z, with an AGC target of 1e6, a maximum IT of 50 ms for the first stage, and a single scan range. Dynamic exclusion was set to 30.0 s. MS/MS spectra were searched against the nonredundant International Protein Index Arabidopsis sequence database v3.85 using the MASCOT engine (Matrix Science, London, UK; version 2.2). The following options were employed for protein identification: peptide mass tolerance = 20 ppm, MS/MS tolerance = 0.1 Da, Enzyme = Trypsin, Missed cleavage = 2, Fixed modification: Carbamidomethyl (C), Variable modification: Oxidation (M).

### Immunofluorescence

HGC-27 cells were fixed with methanol at 4 ℃ for 30 min followed by incubation with anti-PHB2 mouse antibodies (1:50, Proteintech, Wuhan, China) and anti-SHIP2 rabbit antibodies (1:50, CST, Beverly, MA, USA) at 4 ℃ overnight. After blocking the cells with goat serum at room temperature for 30 min, samples were then incubated with Alexa Flour 488 anti-rabbit IgG antibodies and Alexa Flour594 anti-mouse IgG antibodies (1:200, Proteintech, Wuhan, China) for 2 h. Finally, DAPI staining was used to determine the nuclei (Beyotime Biotechnology, Shanghai, China). Images were captured using the ZEISS LSM880 confocal laser scanning microscope (Carl Zeiss, Oberkochen, Germany).

### GST pull-down

PHB1 and PHB2 cDNA sequences were synthesized using specific primers and subcloned into the pGEX-5X-3 vector. The details of specific primers used for synthesis are provided in Supplementary Table 1. GST-PHB1 and GST-PHB2 proteins were purified from BL21 Rosetta (DE3) cells using GST beads (Beyotime Biotechnology, Shanghai, China). tGFP-SHIP2 protein was incubated and rotated with GST, GST-PHB1, or GST-PHB2, respectively, at 4 °C for 4 h, and then incubated with GST beads for an additional 4 h at 4 °C. After washing the beads four times, the supernatants were added SDS buffer and boiled at 98 ℃ for 10 min, and the protein samples were then analyzed using Western blot analysis.

### MTT

4 × 10^3^ cells were seeded in each well of a 96-well plate with 200 μl of media and allowed to grow for 24, 48, 72, and 96 h. After adding 20 μl of MTT (5 mg/ml), the cells were further incubated for 4 h and the absorbance was measured at 490 nm using a microplate reader (USCN KIT INC, Wuhan, China).

### Colony formation

2 × 10^3^ cells were seeded into a six-well plate and maintained in media, with medium replacement every 3 days. After around 14 days, the cells were fixed with methanol and stained with 0.1% crystal violet (Beyotime Biotechnology, Shanghai, China) at room temperature for 30 min. The colonies were counted and imaged using the ImageJ system.

### Soft agar colony formation

A base agar was constituted by adding 1.2% agar to 2 × RPMI 1640 + 20% FBS, while the top agar was constituted by adding 0.7% agar to 2 × RPMI 1640 + 20% FBS, which was kept at 40 °C in a water bath. Next, 5 × 10^3^ cells were added to the top agar and mixed, and then seeded into a six-well plate. The plates were incubated at 37 °C with 5% CO_2_ for 14 days, and the colonies were stained with 0.005% crystal violet (Beyotime Biotechnology, Shanghai, China) at room temperature for 30 min. The colonies were counted using a microscope.

### Subcellular fractionation

After incubating cells to hypotonic Buffer A (containing 10 mM Hepes pH 7.9, 10 mM KCl, 0.1 mM EDTA, 0.1 mM EGTA, 1 mM DTT, 0.15% Triton X-100, EDTA-free Protease Inhibitor Cocktail) on ice for 10 min, the samples were subjected to centrifugation at 3000 × g for 10 min, and the resultant supernatant was collected as the cytosolic fraction. The pellets were rinsed twice with cold hypotonic buffer A, and the nuclear proteins were extracted by treating with Buffer B (containing 20 mM Hepes pH 7.9, 400 mM NaCl, 1 mM EDTA, 1 mM EGTA, 1 mM DTT, 0.5% Triton X-100, EDTA-free Protease Inhibitor Cocktail) on ice for 15 min. The cytosolic and nuclear fractions were then subjected to analysis by qPCR or Western blotting.

### BrdU cell proliferation assay

5 × 10^3^ cells were seeded into a 96-well plate and incubated at 37 °C with 5% CO2 for 24 h. Next, 1 × BrdU solution was added to the plate wells and the cells were incubated for an additional 4 h. The cells were then fixed with Fixing/Denaturing Solution at room temperature for 30 min. After adding 1 × detection antibody solution, 1 × HRP-conjugated secondary antibody solution, TMB Substrate, and STOP Solution in that order, the results were read by measuring the absorbance at 450 nm using a microplate reader (USCN KIT INC, Wuhan, China).

### Xenograft mouse models

5 × 10^6^ suspended cells with sh-PHB2, sh-scramble, and/or Vector alone, myr-Akt were injected subcutaneously into the posterior flanks of female athymic BALB/c nude mice aged 5 weeks that were grown under specific pathogen-free conditions and handled according to protocols provided by Gempharmatech (Nanjing, China). Tumour volumes were measured every 3 days by calculating tumour volume (mm^3^) = 1/2 × tumour length(mm) × tumour width^2^ (mm^2^). After 20 days post-injection, mice were euthanized, tumours were weighed, and H&E staining was performed on paraffin-embedded, formalin-fixed tissues. IHC assays were performed to detect Ki67 protein expression. Studies using animals were approved by the Animal Ethics Review Committee of Anhui Medical University (20190339).

### Statistical analysis

Data were presented as mean ± Standard Error of Mean (SEM), and sample sizes (n) were indicated for each statistical analysis. Statistical differences were analyzed by two-tailed Student’s t-test, one/two-way ANOVA followed by Tukey’s multiple comparison or two-way ANOVA followed by Šídák’s multiple comparisons using GraphPad Prism. *P* values less than 0.05 were statistically significant.

## Results

### PHB2 upregulation is associated with poor patient outcomes in GC

**Fig. 1:**
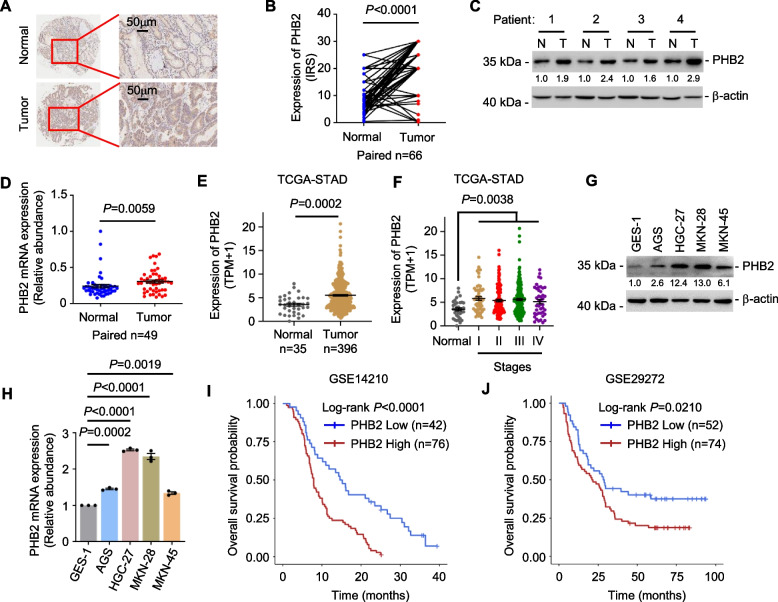
PHB2 upregulation is associated with poor patient outcomes in GC. **A**, **B** Representative images and quantitation of IHC analysis of PHB2 protein expression in 66 pairs of human gastric tumour tissues and corresponding adjacent normal gastric tissues. Scale bar, 50 μm. IRS: Immunoreactive score. Two-tailed Student’s t-test. **C**, **D** Western blotting (**C**) and qRT-PCR (**D**) analysis of PHB2 expression from 49 gastric tumour (T) patient samples and paired adjacent normal (N) gastric tissues. Data are representatives or mean ± SEM, two-tailed Student’s t-test. **E** PHB2 was upregulated in GC compared with normal gastric tissues as revealed by analysis of the STAD data in TCGA dataset. Data are mean ± SEM, two-tailed Student’s t-test. **F** The mRNA expression levels of PHB2 among different stages of GC tissues derived from the TCGA dataset. Data are mean ± SEM, one-way ANOVA followed by Tukey’s multiple comparison. **G**, **H** Western blotting (**G**) and qRT-PCR (**H**) analysis of PHB2 in a panel of GC cell and normal gastric epithelial cell line GES-1. Data are representatives or mean ± SEM; *n* = 3 independent experiments, one-way ANOVA followed by Tukey’s multiple comparison. **I**, **J** Kaplan–Meier analysis of the probability of overall survival of GC patients derived from the GEO datasets (GSE14210, GSE29272) using the optimal of PHB2 levels as the cut-off

In order to evaluate the clinical relevance of PHB2 in the context of GC, we examined the expression of PHB2 using IHC in a GC TMA including 66 paired human gastric tumour (T) tissues and corresponding adjacent normal (N) gastric tissues (Supplementary Table 3). PHB2 expression levels were significantly increased in GC tissues compared to adjacent normal gastric tissues (Fig. 1A, B). Consistent with our initial observations, PHB2 protein and mRNA levels were both increased in 49 GC clinical samples compared with paired normal gastric tissues (Fig. 1C, D). Additionally, PHB2 mRNA levels were analysed in the Stomach Adenocarcinoma (STAD) dataset obtained from the Cancer Genome Atlas (TCGA) through RNA-sequencing (RNA-seq) analysis. Our findings revealed a significant upregulation of PHB2 in GC compared to normal tissues, with no significant variation among different stages of GC (Fig. 1E, F). Moreover, PHB2 protein and mRNA levels were highly expressed in a panel of GC cell lines in comparison to a normal gastric mucosa epithelial cell line GES-1 (Fig. 1G, H).

Furthermore, we employed the Kaplan–Meier plotter analysis on the GEO datasets (GSE14210, GSE29272), which demonstrated a significant correlation between elevated levels of PHB2 and poorer survival outcomes in GC patients (Fig. 1I, J). Subsequently, we investigated the association between PHB2 expression and various clinicopathological features of GC patients. However, our analysis did not reveal any significant correlation between PHB2 expression and gender, age, or other clinicopathological characteristics (Supplementary Table 4).

### PHB2 promotes GC cell proliferation and tumorigenicity

**Fig. 2:**
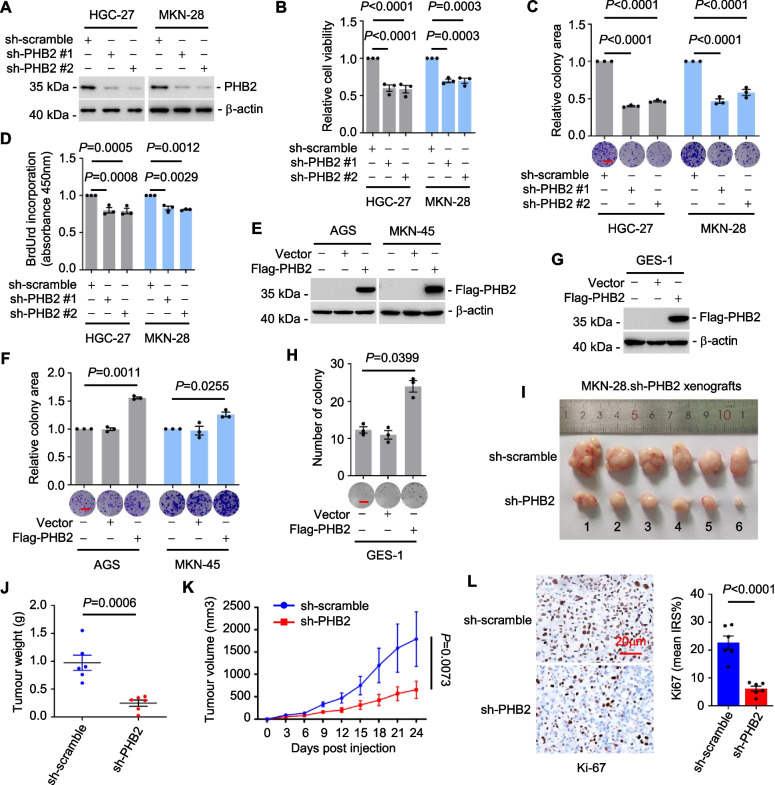
PHB2 promotes GC cell proliferation and tumorigenicity. **A-D** shRNA silencing of PHB2 (**A**) attenuated GC cell proliferation as shown in MTT assays (**B**), clonogenic assays (**C**) and 5-bromo-2′-deoxyuridine (BrdU) incorporation (**D**). Data are representatives or mean ± SEM; *n* = 3 independent experiments, one-way ANOVA followed by Tukey’s multiple comparison. Scale bar, 1 cm. **E**, **F** PHB2 overexpression (**E**) promoted GC cell proliferation as shown in clonogenic assays (**F**). Data are representatives or mean ± SEM; *n* = 3 independent experiments, two-tailed Student’s t-test. Scale bar, 1 cm. **G**, **H** Overexpression of PHB2 (**G**) enhanced anchorage-independent growth of normal gastric epithelial cell line GES-1 (**H**). Data are representatives or mean ± SEM; *n* = 3 independent experiments, two-tailed Student’s t-test. Scale bar, 1 cm. **I-K** Representative photographs (**I**), tumour weight (**J**) and growth curves (**K**) of MKN-28.sh-scramble and MKN-28.sh-PHB2 xenografts in nu/nu mice. Data are representatives or mean ± SEM; *n* = 6 mice per group, two-tailed Student’s t-test (**J**), two-way ANOVA followed by Šídák’s multiple comparisons test (**K**). **L** Representative microscopic photographs (left panel), and quantitation of IHC staining for Ki67 (right panel) in FFPE sections of MKN-28.sh-scramble and MKN-28.sh-PHB2 xenografts. Data are representatives or mean ± SEM; *n* = 6 mice per group, two-tailed Student’s t-test. Scale bar, 20 μm. IRS: Immunoreactive score

To evaluate the functional significance of PHB2 upregulation, two individual short hairpin RNAs (shRNAs) were employed to silence PHB2 expression in HGC-27 and MKN-28 cell lines (Fig. 2A), which exhibited relatively high PHB2 expression levels among a panel of GC cell lines (Fig. 1G). ShRNA knockdown of PHB2 significantly decreased GC cell proliferation using MTT cell viability analysis, clonogenic assays, and BrdU incorporation assays (Fig. 2B-D), consistently indicating that PHB2 promotes GC cell proliferation. Contrariwise, overexpression of PHB2 in AGS and MKN-45 cell lines, which expressed relatively low levels of endogenous PHB2 (Fig. 1G), promoted GC cell proliferation (Fig. 2E, F). Moreover, we observed that PHB2 overexpression enhanced anchorage-independent growth in normal GES-1 cells (Fig. 2G, H), indicating that PHB2 potentially induce the oncogenic transformation of normal gastric mucosa epithelial cells.

To further investigate the role of PHB2 in vivo, we established MKN-28.sh-PHB2 sublines with stable shRNA knockdown of PHB2 expression and investigated tumour growth in nu/nu mice. These mice were xenografted with MKN-28.sh-PHB2 and MKN-28.sh-scramble control cells through subcutaneous transplantation, which led to a significant reduction in tumour growth in MKN-28.sh-PHB2 compared to MKN-28.sh-scramble control groups (Fig. 2I-K), accompanied by a decrease in cell proliferation within the tumours as indicated by the proportion of Ki67-expressing cells (Fig. 2L). These findings support the notion that PHB2 plays a role in promoting GC cell proliferation and tumorigenicity.

### PHB2 binds to SHIP2 in the cytoplasm of GC cells

**Fig. 3:**
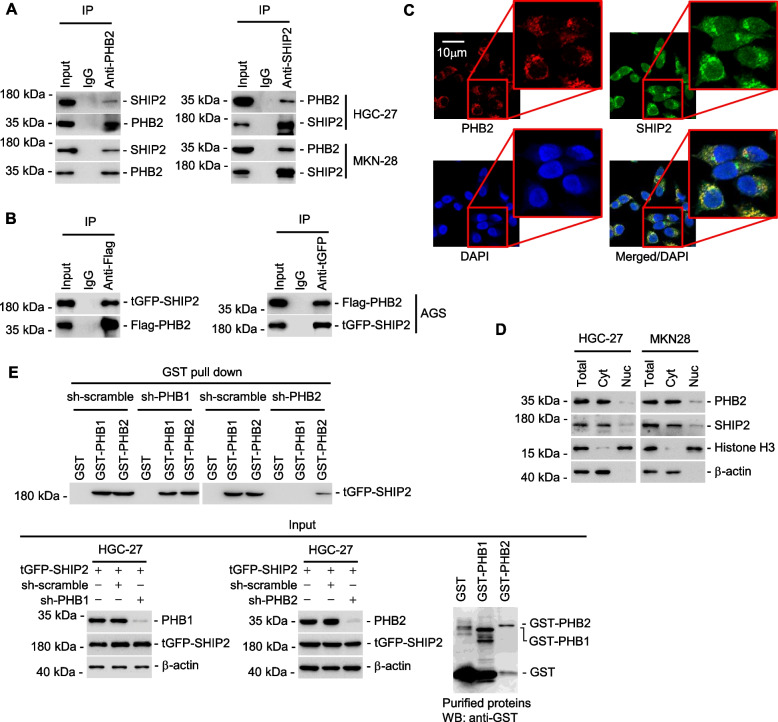
PHB2 binds to and co-localizes with SHIP2 in the cytoplasm of GC cells. **A** Endogenous PHB2 and SHIP2 were co-precipitated with each other in HGC-27 and MKN-28 cells. **B** Exogenous Flag-PHB2, and tGFP-SHIP2 were co-precipitated with each other in AGS cells. **C** Representative immunofluorescence photographs of PHB2 and SHIP2 co-localization in HGC-27 cells. Scale bar, 10 μm. **D** Western blotting showing subcellular localization of PHB2 and SHIP2. Cyt: cytoplasm; Nuc: nucleus. β-actin: Cyt marker; Histone H3: Nuc marker. **E** tGFP-SHIP2 was co-pulled down by recombinant GST-PHB1 or GST-PHB2, which was however diminished by PHB2 knockdown. Data are representatives of three independent experiments

To further investigate the mechanism by which PHB2 enhances the proliferation of GC cells, we employed immunoprecipitation plus mass spectrometry (IP-MS) analysis to identify potential novel protein(s) that interact with PHB2 in HGC-27 cells. Among the top candidates, SHIP2 was identified as one protein co-precipitated with PHB2 (Supplementary Table 5). The binding between PHB2 and SHIP2 was validated through endogenous co-immunoprecipitation in HGC-27 and MKN-28 cell lines, as well as exogenous co-immunoprecipitation in the AGS cell line (Fig. 3A, B). Moreover, immunofluorescence staining and subcellular fractionation assays revealed that PHB2 and SHIP2 co-localized in the cytoplasm of GC cells (Fig. 3C, D), providing the necessary spatial conditions for their interaction. Subsequently, the specific binding between SHIP2 and PHB2 was demonstrated by the co-pulldown of tGFP-tagged SHIP2 with GST-tagged PHB2, while no binding was observed with GST-tagged PHB1, the homologue of PHB2 (Fig. 3E).

Remarkably, PHB2 showed a direct interaction with SHIP2 in HGC-27 cells with stable knockdown of PHB1, as evidenced by GST pull-down assay (Fig. 3E). In contrast, no interaction was observed between PHB1 and SHIP2 in the same cells with PHB2 knocked down (Fig. 3E), suggesting that SHIP2 specifically binds to PHB2 but not PHB1. These findings were further supported by co-immunoprecipitation (co-IP) assays. In particular, co-IP experiments demonstrated that Flag-tagged PHB2 co-precipitated with tGFP-tagged SHIP2 in HGC-27 cells with stable PHB1 knockdown (Supplementary Fig. 1A). Conversely, HA-tagged PHB1 did not co-precipitate with tGFP-tagged SHIP2 in HGC-27 cells with stable PHB2 knockdown (Supplementary Fig. 1B). Overall, the results provide compelling evidence that SHIP2 directly interacts with PHB2 but not PHB1 in HGC-27 cells.

**Fig. 4:**
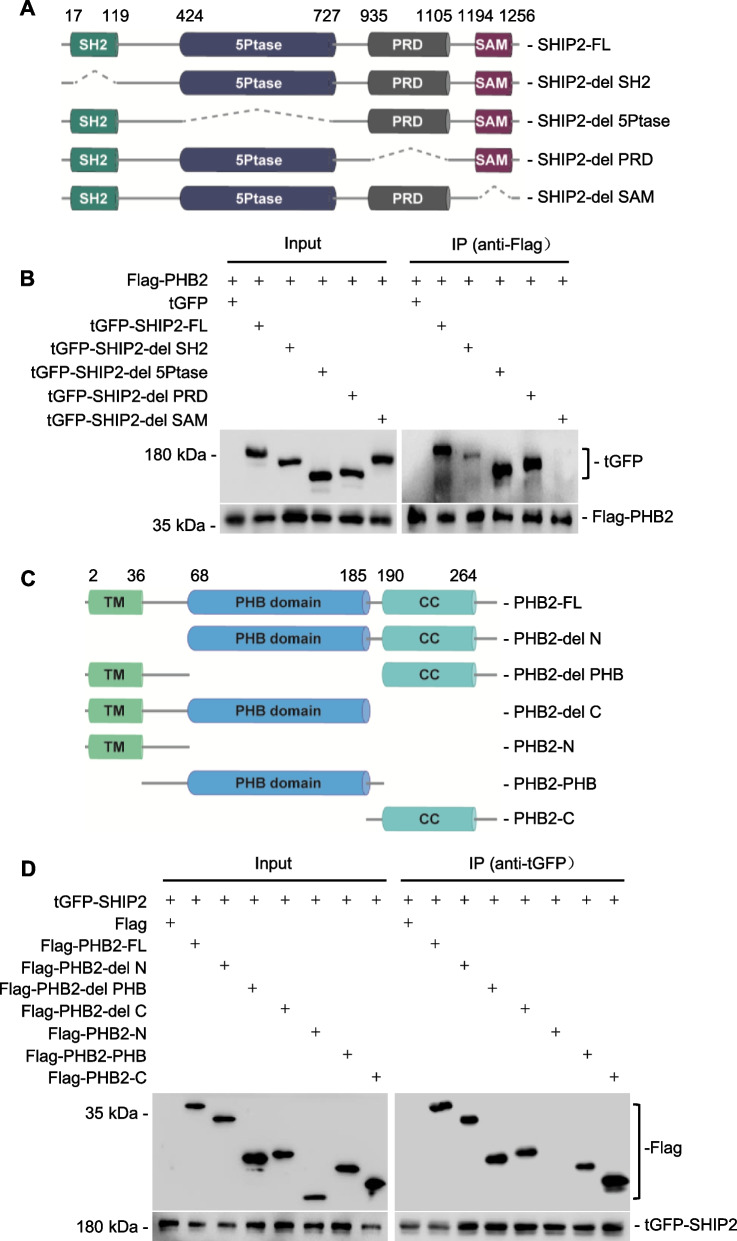
The SAM domain of SHIP2 and all regions of PHB2 except for the N-terminal are required for the interaction between PHB2 and SHIP2. **A** Schematic illustration of full-length SHIP2 (SHIP2-FL) and its corresponding truncates used in mapping experiments. **B** Deletion-mapping experiments showing that the SAM domain of SHIP2 is indispensable for its interaction with PHB2 in HEK293T cells. **C** Schematic illustration of full-length PHB2 (PHB2-FL) and the PHB2 mutants. **D** Deletion-mapping experiments showing that the N-terminal region of PHB2 is dispensable for its interaction with SHIP2 in HEK293T cells. Data are representatives of three independent experiments

In order to identify the specific regions of SHIP2 and PHB2 responsible for their interaction, we conducted deletion-mapping experiments. Our findings revealed that the SHIP2 fragment corresponding to the SAM domain is essential for its binding to PHB2 (Fig. 4A, B). Additionally, deletion-mapping experiments with PHB2 mutants determined that the N-terminal region of PHB2 is dispensable for its interaction with SHIP2 (Fig. 4C, D). Therefore, we concluded that the SAM domain of SHIP2 and all regions of PHB2 except for the N-terminal, play critical roles in their interaction.

### PHB2 promotes SHIP2 protein degradation through ubiquitination

**Fig. 5:**
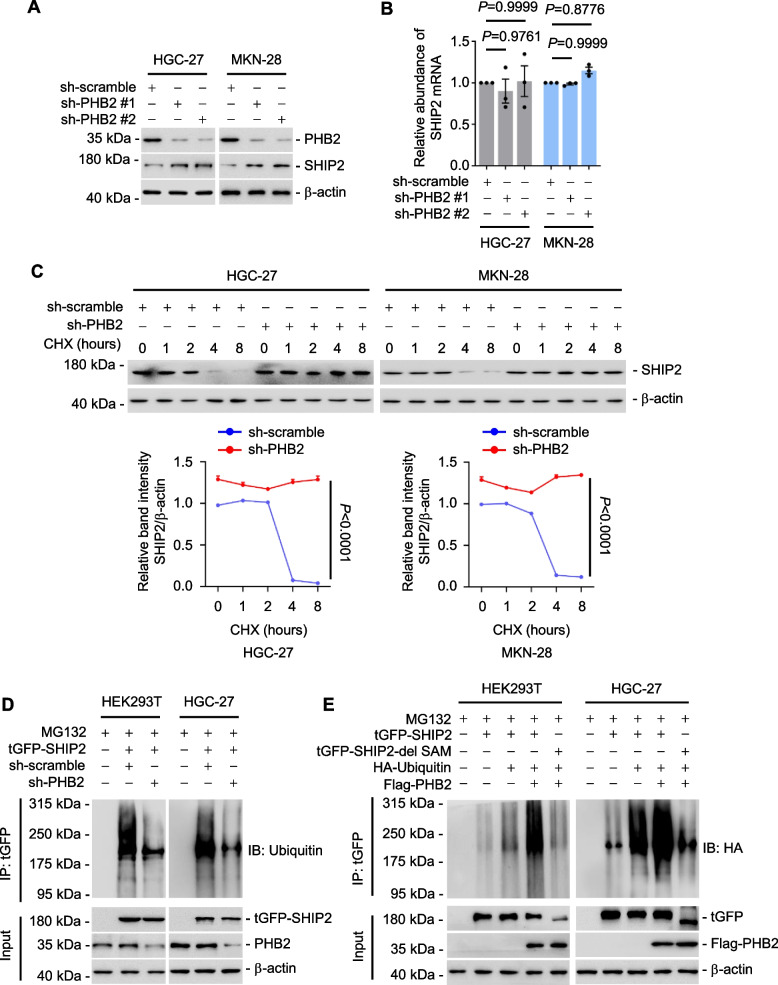
PHB2 promotes SHIP2 protein degradation through ubiquitination. **A**, **B** Western blotting (**A**) and qRT-PCR analysis (**B**) of SHIP2 expression in HGC-27.sh-PHB2 and MKN-28.sh-PHB2 cells. Data are representatives or mean ± SEM; *n* = 3 independent experiments, one-way ANOVA followed by Tukey’s multiple comparison. **C** Silencing of PHB2 prolonged the half-life time of SHIP2 protein in cycloheximide (CHX)-chase assays. Data are representatives or mean ± SEM; *n* = 3 independent experiments, two-way ANOVA followed by Šídák’s multiple comparisons test. CHX: 10 μg/ml. **D** Silencing of PHB2 reduced overexpressed exogenous SHIP2 ubiquitination. MG132:10 μM. Data shown represent three independent experiments. **E** Overexpression of PHB2 enhanced the ubiquitination of exogenous full-length SHIP2 but not tGFP-SHIP2-del SAM mutant SHIP2. MG132: 10 μM. Data shown represent three independent experiments

Our study revealed that PHB2 is frequently expressed at higher levels (Fig. 1A-F), whereas SHIP2 is usually downregulated in GC tissues compared to adjacent normal gastric tissues [25], suggesting a potential negative regulatory relationship between PHB2 and SHIP2 in GC. Indeed, knockdown of PHB2 by shRNA significantly increased protein levels of SHIP2 (Fig. 5A) without affecting mRNA levels (Fig. 5B). In contrast, overexpression of Flag-tagged PHB2 in HGC-27 and MKN-28 cells significantly reduced endogenous SHIP2 protein levels in a dose-dependent manner (Supplementary Fig. 2A). Of note, PHB2 accelerated the turnover rate of the SHIP2 protein (Fig. 5C), indicating the role of PHB2 in the regulation of SHIP2 protein stability.

In order to examine the influence of ubiquitination on SHIP2 protein stability, we conducted experiments to investigate whether PHB2 facilitates the degradation of SHIP2 through ubiquitination. Co-transfection of tGFP-SHIP2 and His-Ubiquitin confirmed the presence of ubiquitinated tGFP-SHIP2 in GC cells (Supplementary Fig. 2B). Strikingly, knockdown of PHB2 using shRNA resulted in a significant reduction in SHIP2 ubiquitination (Fig. 5D). Conversely, overexpression of PHB2 led to an increase in the ubiquitination of full-length SHIP2 but not of the SHIP2 variant lacking the SAM domain, which is essential for the interaction between SHIP2 and PHB2 (Fig. 5E). Collectively, these findings indicate that the binding of SHIP2 to PHB2 plays a critical role in SHIP2 ubiquitination.

### PHB2 enhances the binding between SHIP2 and its E3 ligase NEDD4

**Fig. 6:**
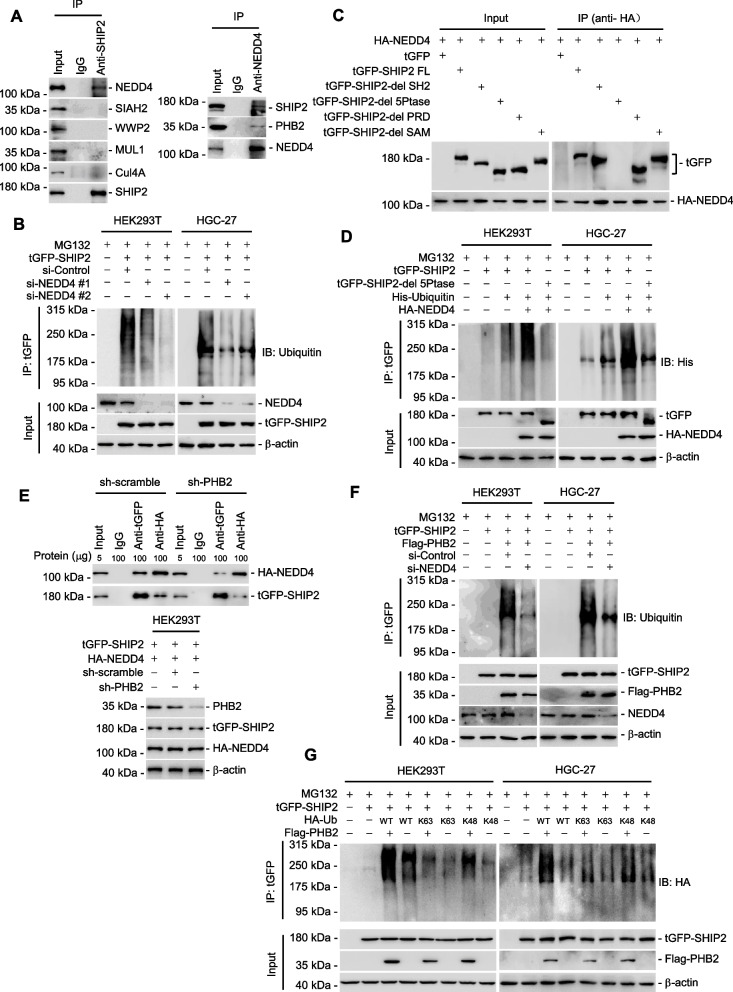
PHB2 induces the ubiquitination degradation of SHIP2 by enhancing the interaction between NEDD4 and SHIP2. **A** Co-IP assays showing that NEDD4 was specifically co-precipitated with SHIP2 and PHB2 in HGC-27 cells. E3 ligases SIAH2, WWP2, MUL1 and Cul4A were used as negative controls. Data shown represent three independent experiments. **B** SiRNA knockdown of NEDD4 decreased the ubiquitination of SHIP2. MG132: 10 μM. **C** Deletion-mapping experiments showing that NEDD4 was precipitated with the 5-Ptase domain of SHIP2 but not other SHIP2 domains in HEK293T cells. **D** Overexpression of NEDD4 increased the ubiquitination full-length SHIP2 but not tGFP-SHIP2-del 5Ptase in HEK293T and HGC-27 cells. MG132:10 μM. **E** Silencing of PHB2 reduced the interaction between SHIP2 and NEDD4 in HEK293T cells. **F** Silencing of NEDD4 abolished the ubiquitination of SHIP2 caused by PHB2 overexpression. **G,** Overexpression of PHB2 mainly increased K48-linked polyubiquitination of SHIP2. MG132:10 μM. Data are representatives of three independent experiments

To further understand the mechanism responsible for the PHB2-mediated ubiquitination degradation of SHIP2 in GC cells, we investigated whether the degradation of SHIP2 by PHB2 was facilitated through E3 ligases-mediated ubiquitination. We used immunoprecipitation followed by mass spectrometry to analyze the proteins that interacted with SHIP2, with a specific focus on E3 ligases. Among the proteins identified, NEDD4 emerged as the sole E3 ligase candidate (Supplementary Table 6), and its binding to SHIP2 was confirmed using co-IP assays in HGC-27 cells (Fig. 6A). Conversely, no interaction was observed between SHIP2 and other notable E3 ligases, such as SIAH2, WWP2, MUL1, and Cul4A (Fig. 6A) [28]. This underscores the specificity of the SHIP2-NEDD4 binding and suggests that the interaction between SHIP2 and its E3 ligase may occur in a tissue-specific manner. Furthermore, the silencing of NEDD4 by siRNA resulted in a notable reduction in the ubiquitination of SHIP2 (Fig. 6B), providing compelling evidence that NEDD4 mediates the ubiquitination of SHIP2.

To further identify the region of SHIP2 responsible for its interaction with NEDD4 and determine whether this region plays a crucial role in the NEDD4-mediated ubiquitination of SHIP2, we conducted deletion-mapping experiments using various SHIP2 truncates (Fig. 6C). The results of these experiments unveiled that the 5Ptase domain of SHIP2 is indispensable for its interaction with NEDD4 (Fig. 6C). Moreover, NEDD4 overexpression led to a significant increase in the ubiquitination of SHIP2 but not SHIP2 mutant lacking 5Ptase domain (Fig. 6D). These findings strongly suggest that NEDD4 facilitates the ubiquitination of SHIP2 by specifically interacting with the 5Ptase domain of SHIP2.

While SHIP2 levels decrease, PHB2 levels increase in GC tissues (Fig. 1A-F) [25], prompting an investigation into the expression of the E3 ligase NEDD4 for SHIP2 in GC tissues. Our analysis of TCGA data revealed that NEDD4 expression was elevated in GC tissues compared to adjacent normal gastric tissues (Supplementary Fig. 3A). This finding was further supported by the confirmation of increased NEDD4 mRNA and protein levels in a panel of GC cell lines compared to normal GES-1 cells (Supplementary Fig. 3B, C) and in randomly selected four pairs of GC tissues and adjacent normal gastric tissues (Supplementary Fig. 3D). Interestingly, silencing PHB2 did not exert a significant effect on NEDD4 protein and mRNA levels (Supplementary Fig. 3E, F), advocating that the PHB2-mediated induction of SHIP2 ubiquitination degradation does not occur through the regulation of NEDD4 expression.

To investigate whether PHB2 affected SHIP2 ubiquitination via the recruitment of NEDD4, we conducted co-immunoprecipitation assays and found that silencing PHB2 disturbed the interaction between NEDD4 and SHIP2 (Fig. 6E), indicating that PHB2 facilitated SHIP2 ubiquitination by promoting the interaction between NEDD4 and SHIP2. Further supporting this notion, our findings revealed that overexpression of PHB2 promoted SHIP2 ubiquitination, which was subsequently reversed upon knockdown of NEDD4 (Fig. 6F). To determine the specific ubiquitin site on SHIP2, we co-transfected HA-Ub-WT, HA-Ub-K63, or HA-Ub-K48 along with Flag-PHB2 and tGFP-SHIP2 into HEK293T and HGC-27 cells. Our results indicated that PHB2 majorly enhanced the K48-linked polyubiquitylation of SHIP2 (Fig. 6G). Taken together, our findings demonstrate that PHB2 induces the degradation of SHIP2 through ubiquitination by augmenting the interaction between the E3 ligase NEDD4 and SHIP2.

### PHB2-mediated SHIP2 degradation leads to Akt activation and GC proliferation

**Fig. 7:**
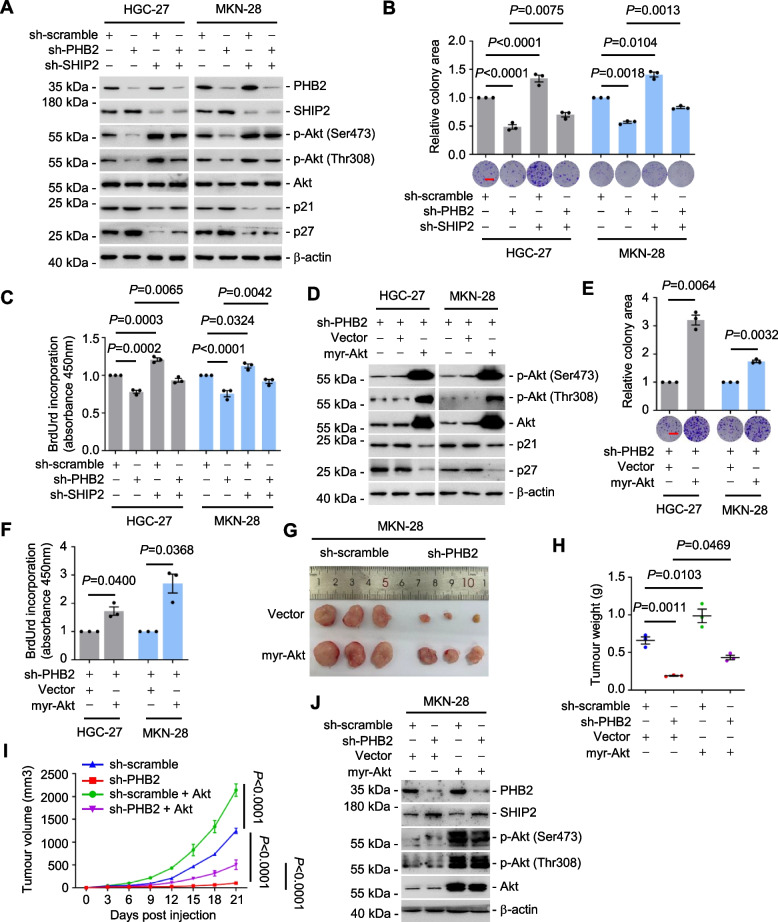
PHB2-mediated SHIP2 degradation leads to Akt activation and GC proliferation. **A** Knockdown of PHB2 increased SHIP2 protein expression and diminished Akt activation, which was reversed by co-knockdown of SHIP2. Data shown represent three independent experiments. **B**, **C** Knockdown of PHB2 inhibited GC cell proliferation, which was abolished by co-knockdown of SHIP2 as shown in clonogenic assays (**B**) and BrdU incorporation (**C**). Data are representatives or mean ± SEM; *n* = 3 independent experiments, one-way ANOVA followed by Tukey’s multiple comparison. Scale bar, 1 cm. **D-F,** Overexpression of myr-Akt reversed the inhibition of Akt signaling (**D**) and GC cell proliferation caused by silencing of PHB2 as shown in clonogenic assays (**E**) and BrdU incorporation (**F**). Data are representatives or mean ± SEM; *n* = 3 independent experiments, two-tailed Student’s t-test. Scale bar, 1 cm. **G-I** Representative Photographs (**G**), tumour weight (**H**) and growth curves (**I**) of MKN-28.sh-scramble and MKN-28.sh-PHB2 xenografts in nu/nu mice with or without co-transduction of myr-Akt. Data are representatives or mean ± SEM; *n* = 3 mice per group, one-way ANOVA followed by Tukey’s multiple comparison (**H**), two-way ANOVA followed by Tukey’s multiple comparison (**I**). **J** Western blotting showing the expression of PHB2, SHIP2 and Akt in GC cells isolated from MKN-28.sh-scramble and MKN-28.sh-PHB2 xenografts harvested from mice treated as described in **G**. Data are representatives of three independent experiments

**Fig. 8:**
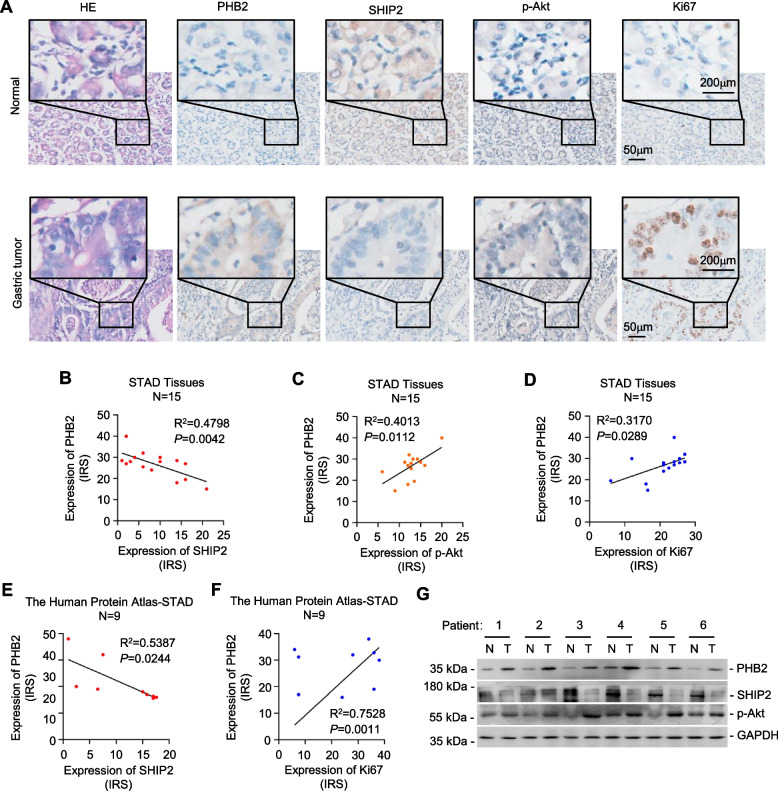
PHB2 expression is negatively correlated with SHIP2 expression, and positively correlated with p-Akt and Ki67 expression in GC. **A** representative photograph showed the expression of PHB2, SHIP2, p-Akt, and Ki67 in GC tissues and adjacent normal tissues examined by IHC staining (*n* = 15). Scale bar: 200 μm. **B-D** the expression correlations between PHB2 and SHIP2 (**B**), PHB2 and p-Akt (**C**), and PHB2 and Ki67 (**D**) depicted in A were analysed using using Pearson’s correlation coefficient test. *n* = 15 clinical samples. **E**, **F** the expression correlations between PHB2 and SHIP2 (**E**), and PHB2 and Ki67 (**F**) in GC tissues from the Human Protein Atlas dataset (https://www.proteinatlas.org) were analysed using Pearson’s correlation coefficient test. *n* = 9 clinical samples. **G** Western blotting analysis of PHB2, SHIP2, p-Akt, and GAPDH protein expression in gastric tumour (T) patient samples and paired adjacent normal (N) gastric tissues. Data are representatives

**Fig. 9:**
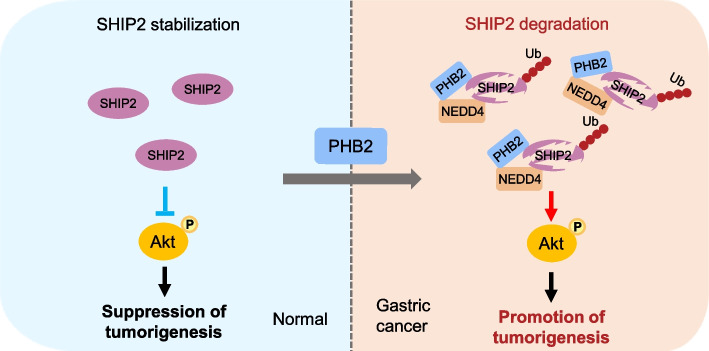
Schematic illustrating the role of PHB2 in regulating GC tumorigenesis. SHIP2 acts as a negative regulator of phosphoinositide 3-kinase (PI3K) and insulin signaling. In GC cells, high expression of PHB2 enhanced the interaction between NEDD4 and SHIP2, which triggers the ubiquitination degradation of SHIP2, consequently leading to the activation of Akt and promoting GC proliferation and tumorigenesis

In our previous study [25, 26], we revealed that SHIP2 repressed cell proliferation and motility in GC by inhibiting the PI3K/Akt signaling pathway. Our results revealed that silencing PHB2 led to increased SHIP2 expression levels and diminished Akt activation in both GC cell lines (Supplementary Fig. 4A) and in the xenografted mouse model (Supplementary Fig. 4B). This effect was reversed upon silencing SHIP2 (Fig. 7A), leading to augmented GC cell proliferation, as evidenced by clonogenic assays (Fig. 7B) and BrdU incorporation (Fig. 7C). These findings highlighted the critical role of SHIP2 as a key regulator in the PHB2-driven cell proliferation process in GC. To further support the observations, overexpression of a constitutively active Akt (myr-Akt) in HGC-27.sh-PHB2 and MKN-28.sh-PHB2 cells resulted in hyperactivation of Akt (Fig. 7D). Remarkably, this hyperactivation of Akt rescued the antiproliferative effect of PHB2 silencing (Fig. 7E, F). Moreover, we substantiated the involvement of PI3K/Akt signaling in the PHB2-mediated GC tumorigenesis by transplanting MKN-28.sh-PHB2 cells co-transfected with myr-Akt into nu/nu mice (Fig. 7G, H). Our results demonstrated that the expression of myr-Akt effectively restored tumour growth (Fig. 7G-I), accompanied by Akt reactivation in mouse xenografts carrying sh-PHB2 (Fig. 7J). In GC patient samples, the expression of PHB2 exhibited a negative correlation with SHIP2 in GC tissues (Fig. 8A, B), but this correlation was not observed in adjacent normal tissues (Supplementary Fig. 4C). Conversely, PHB2 expression demonstrates a positive correlation with both p-Akt (indicative of Akt activation) and Ki67 (a marker for cell proliferation) expression in GC tissues (Fig. 8A-D). These findings were substantiated by the analysis of clinical data from the Human Protein Atlas-STAD dataset (https://www.proteinatlas.org), confirming the negative correlation between PHB2 and SHIP2, along with the positive correlation between PHB2 and Ki67 (Fig. 8E, F). Furthermore, our clinical samples revealed that elevated PHB2 expression correlates with reduced SHIP2 protein levels, subsequently resulting in the activation of Akt in GC patient tumour tissues in comparison with adjacent normal tissues (Fig. 8G). Taken together, our findings suggest that PHB2 promotes GC cell proliferation by destabilizing SHIP2 protein expression, subsequently leading to Akt activation (Fig. 9).

## Discussion

Being highly conserved proteins, the roles of PHBs in tumour development have ignited intense debates. The question of whether these proteins fuel or suppress cancer has yielded conflicting outcomes made more complex by their varying impacts across distinct tumour types [7, 29–33]. This intricacy underscores the urgent need for meticulous research delving into the mechanisms that govern PHBs’ effects on specific tumours. Moreover, most studies have mainly focused on understanding how PHB1 affects tumours, leaving fewer investigations into the effects of PHB2, especially in GC [15, 34–36]. PHB2 also plays a dual role in various cancer types, acting either as an oncogene or tumour suppressor. In breast cancers, PHB2 exhibits tumour-suppressive functions by inhibiting cell proliferation [17]. Conversely, in NSCLC and hepatocellular carcinoma, PHB2 promotes tumorigenesis by supporting cell survival and migration [10, 12]. The context-dependent nature of PHB2’s role underscores its complexity, necessitating detailed investigations for a comprehensive understanding of its diverse contributions across different cancer types. Nonetheless, our ongoing investigation unveils that, similar to its increased presence in various cancer types, PHB2 is elevated in GC tissues compared to nearby healthy gastric tissues and PHB2 fosters proliferation in GC cells through an innovative pathway. As such, our study furnishes compelling substantiation of PHB2’s oncogenic implications in the context of GC.

PHB2’s functionality exhibits a dependence on its location and its interaction with PHB1 in various contexts [2, 37, 38]. In the mitochondria, PHB1 and PHB2 collaborate to create ring complexes that govern critical processes such as cristae regulation, mitophagy, apoptosis, and cell proliferation [6, 39–41]. Upon penetrating the nucleus, PHB2 is capable of engaging in direct interactions with DNA and RNA, or it can interface with transcription factors such as ER, MyoD, MEF2, and HES1 [42–45]. Notably, research suggests that PHB2 may operate as a transcriptional cofactor due to the direct binding of both PHB1 and PHB2 to the PIG3 promoter DNA [46]. Our study revealed that PHB2 predominantly localizes within the cytoplasm of GC cells and facilitates the degradation of SHIP2, thereby promoting the activation of Akt. Although our data indicates a direct binding between PHB2 and SHIP2, unlike PHB1, further investigation is necessary to ascertain whether PHB2’s role in the cytoplasm of GC cells operates independently of PHB1.

Increasing studies have confirmed that SHIP2 interacts with various proteins, including receptors, cytoskeletal proteins, adaptors, kinases, and phosphatases [47–50]. These interactions play integral roles in a range of important biological processes within the context of cancer. For example, in glioma cells, SHIP2 directly interacts with GTP-bound RhoA, resulting in the promotion of cell polarity and migration [51]. Furthermore, insulin receptor tyrosine kinase substrate (IRTKS) engages with SHIP2, inhibiting its phosphoinositide phosphatase activity [50]. This inhibition subsequently amplifies the activation of the PI3K-Akt signaling pathway, thereby promoting cellular proliferation. However, our previous study demonstrated that SHIP2 is downregulated in GC tissues compared to adjacent normal gastric tissues. This downregulation of SHIP2 contributes to the tumorigenesis and proliferation of GC cells through the activation of Akt [25]. The intricate regulatory mechanisms that govern SHIP2 in cancer cells, in particular, in the context of GC cells are still awaiting comprehensive elucidation. To address this gap, we employed a combination of co-IP and Mass Spectrometry techniques to systematically screen for specific protein binding partners of SHIP2 in GC cells. Our investigation culminated in the identification of NEDD4 as an E3 ligase tasked with orchestrating the ubiquitination and subsequent degradation of SHIP2 within the GC cellular context.

NEDD4, an E3 ubiquitin ligase belonging to the HECT family, can recognize different substrate proteins to catalyze ubiquitination and mediate receptor-mediated endocytosis [52]. Moreover, emerging evidence suggests that NEDD4 plays a key regulatory role in the tumorigenesis process [53]. Consistently, aberrant expression of NEDD4 has been observed in numerous human cancers, including GC, bladder cancer, NSCLC, and breast cancer [54–57]. A study has reported that NEDD4 knockdown dramatically inhibited migration and invasion in GC cells [53]. NEDD4 catalyzes the degradation of its substrate by monoubiquitination of K6 or K27, or by polyubiquitination at K48 and K63, suggesting that NEDD4 plays a variety of regulatory roles through single/multiple ubiquitinations and is thus involved in cellular processes [58–62]. In our study, we found that PHB2 silencing downregulated SHIP2 but did not affect the expression of NEDD4, and the interaction between PHB2 and SHIP2 can also enhance the interaction between SHIP2 and NEDD4, which further primarily enhanced lys-48-linked polyubiquitylation of SHIP2, leading to the degradation of the SHIP2 protein. However, whether this regulation among PHB2, SHIP2, and NEDD4 might be applicable to other types of cancer remains to be defined.

PHB2 holds a pivotal role in organ development, evident from the disruption of organ function upon its loss [9, 63, 64]. Notably, as previously mentioned, PHB2 demonstrates divergent behavior across different cancer types, wherein its overexpression can function as either a tumour suppressor or an oncogene. Given its oncogenic role in the majority of cancer types including GC reported here, PHB2 emerges as a promising target for innovative cancer treatments. Despite the creation of twelve distinct PHB2 modulators, their clinical trial evaluation remains pending [9, 65–67]. This highlights an avenue for forthcoming research and development aimed at curbing PHB2’s crucial impact on cancer, underscoring the potential for future therapeutic breakthroughs.

### Supplementary Information

**Additional file 1: Supplementary Figure 1.** SHIP2 directly interacts with PHB2. **Supplementary Figure 2.** PHB2 regulates the protein expression of SHIP2 through ubiquitination. **Supplementary Figure 3.** PHB2 induces the ubiquitination degradation of SHIP2 by enhancing the interaction between NEDD4 and SHIP2. **Supplementary Figure 4.** PHB2 destabilizes the protein expression of SHIP2, which in turn activates Akt. **Supplementary Table 1.** List of primers. **Supplementary Table 2.** shRNA and siRNA sequences. **Supplementary Table 3.** Gastric cancer tissue array (90 cases/180 cores). **Supplementary Table 4.** Relationship between PHB2 expression and clinicopathologic characteristics of gastric cancer. **Supplementary Table 5.** PHB2-interacting protein candidates identified by immunoprecipitation plus mass spectrometry analysis in HGC-27 cells.

## Data Availability

All data supporting the findings of this study are available from the corresponding author on reasonable request.

## Abbreviations

CHX: Cycloheximide
co-IP: Co-immunoprecipitation
EGFR: Epidermal growth factor receptor
Erα: Estrogen receptor-alpha
ESCC: Esophageal squamous cell carcinoma
GC: Gastric cancer
IHC: Immunohistochemistry
IP-MS: Immunoprecipitation plus mass spectrometry
IQGAP2: IQ motif containing the GTPase-activating protein 2
IRS: Immune Reaction Scores
IRTKS: Insulin receptor tyrosine kinase substrate
NSCLC: Non-small cell lung cancer
PHB1: Prohibitin
PHB2: Prohibitin 2
PI3K: Phosphoinositide 3-kinase
PRD: Proline-rich domain
RACK1: Receptor for activated C kinase 1
RNA-seq: RNA-sequencing
SAM: Sterile alpha motif
SEM: Standard Error of Mean
SHIP2: Src homology 2-containing inositol 5-phosphatase 2
shRNA: Short hairpin RNA
siRNA: Small interfering RNA
SKP2: S-phase kinase-associated protein 2
STAD: Stomach Adenocarcinoma
TCGA: The Cancer Genome Atlas
5-FU: 5-Fluorouracil
