# Supplementary material for: PHB2 promotes SHIP2 ubiquitination via the E3 ligase NEDD4 to regulate AKT signaling in gastric cancer
Source: J Exp Clin Cancer Res. 2024 Jan 11;43:17. doi: 10.1186/s13046-023-02937-1 (PMC10782615; doi:10.1186/s13046-023-02937-1)
Supplement: Supplementary file 1 — Additional file 1: Supplementary Figure 1. SHIP2 directly interacts with PHB2. Supplementary Figure 2. PHB2 regulates the protein expression of SHIP2 through ubiquitination. Supplementary Figure 3. PHB2 induces the ubiquitination degradation of SHIP2 by enhancing the interaction between NEDD4 and SHIP2. Supplementary Figure 4. PHB2 destabilizes the protein expression of SHIP2, which in turn activates Akt. Supplementary Table 1. List of primers. Supplementary Table 2. shRNA and siRNA sequences. Supplementary Table 3. Gastric cancer tissue array (90 cases/180 cores). Supplementary Table 4. Relationship between PHB2 expression and clinicopathologic characteristics of gastric cancer. Supplementary Table 5. PHB2-interacting protein candidates identified by immunoprecipitation plus mass spectrometry analysis in HGC-27 cells. [file 13046_2023_2937_MOESM1_ESM.pdf]

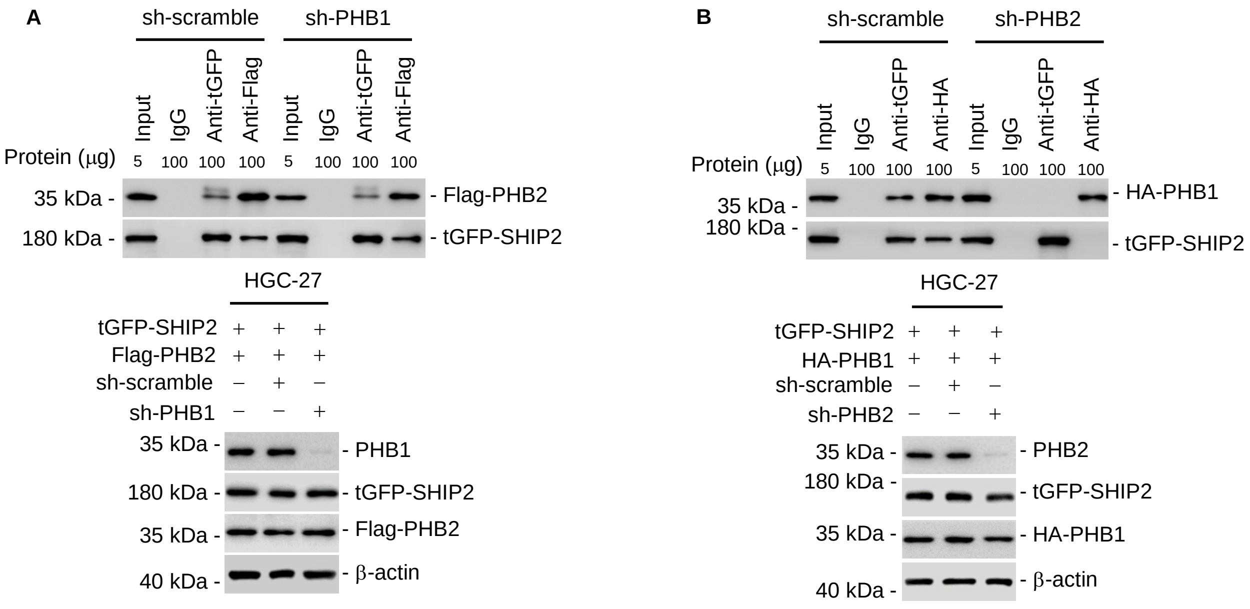

Supplementary Figure 1. SHIP2 directly interacts with PHB2.

A, PHB1 silencing didn't affect the interaction between SHIP2 and PHB2. **B,** PHB2 silencing inhibited the interaction between SHIP2 and PHB1. Data are representatives of three independent experiments.

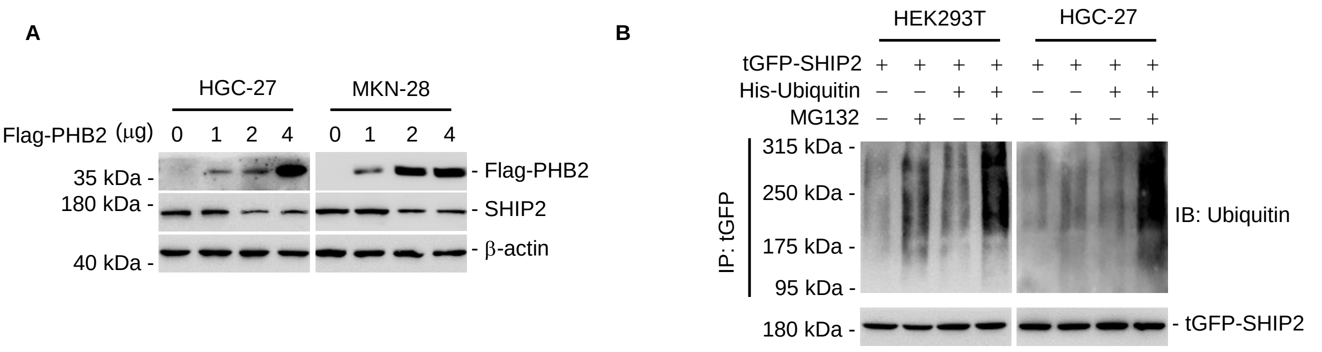

Supplementary Figure 2. PHB2 regulates the protein expression of SHIP2 through ubiquitination.

A, Overexpression of flag tagged PHB2 inhibited endogenous SHIP2 expression in a dose dependent manner. **B,** Co-transfection of tGFP-SHIP2 and His-Ubiquitin confirmed the presence of ubiquitinated tGFP-SHIP2 in GC cells. MG132: 10 μM. Data are representatives of three independent experiments.

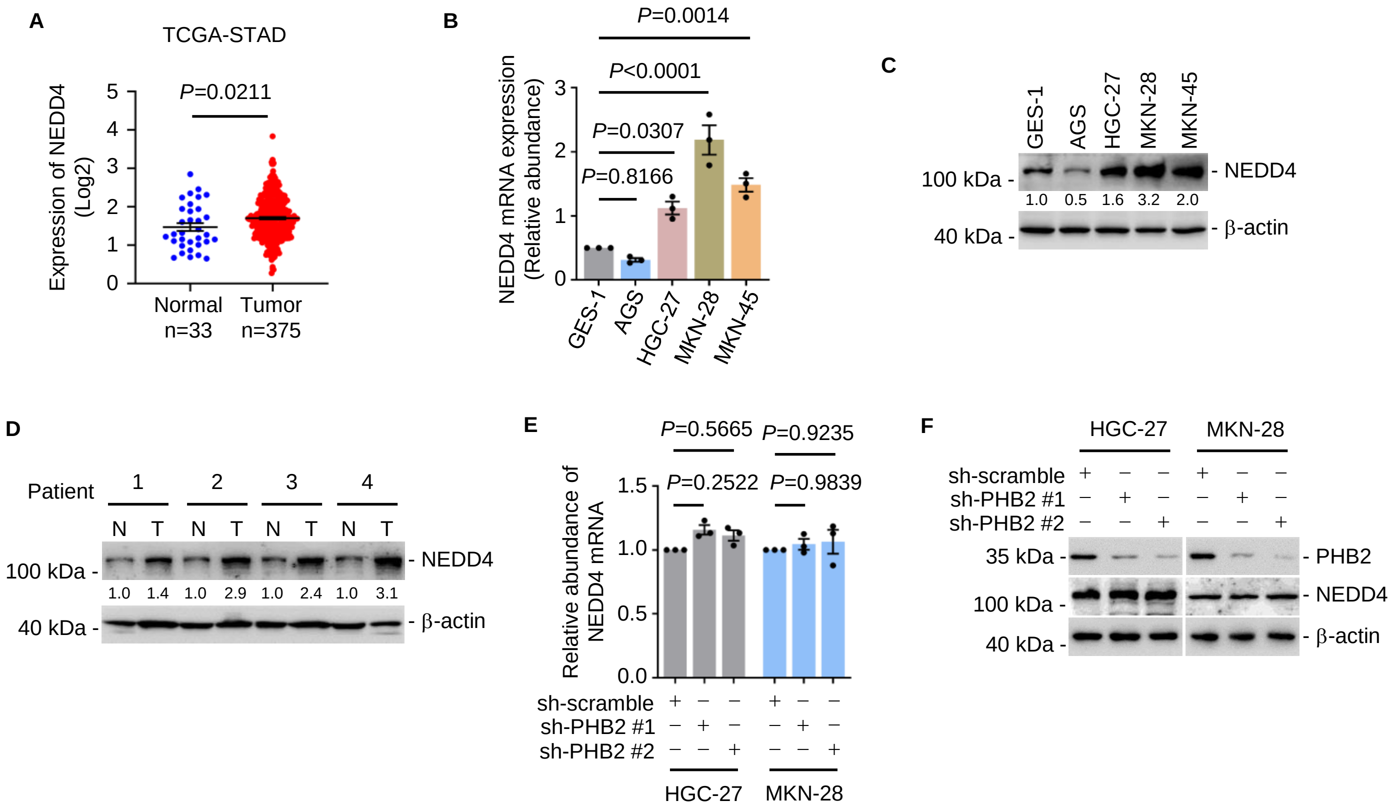

Supplementary Figure 3. PHB2 induces the ubiquitination degradation of SHIP2 by enhancing the interaction between NEDD4 and SHIP2.

A, NEDD4 mRNA expression levels in GC compared with normal gastric tissues in STAD dataset derived from TCGA. Data are mean ± SEM; two-tailed Student's t-test. **B, C,** qRT-PCR (B) and Western blotting (C) analysis of NEDD4 expression in a panel of GC cell lines and normal gastric epithelial cell line GES-1. Data are representatives or mean ± SEM; n =3 independent experiments, one-way ANOVA followed by Tukey's multiple comparison. **D,** Western blotting analysis of NEDD4 expression in GC tumor (T) compared with corresponding paired adjacent normal (N) tissues. Data are representatives of three independent experiments. **E, F,** qRT-PCR (E) and Western blotting (F) analysis of NEDD4 expression after PHB2 knockdown. Data are representatives or mean ± SEM; n =3 independent experiments, one-way ANOVA followed by Tukey's multiple comparison.

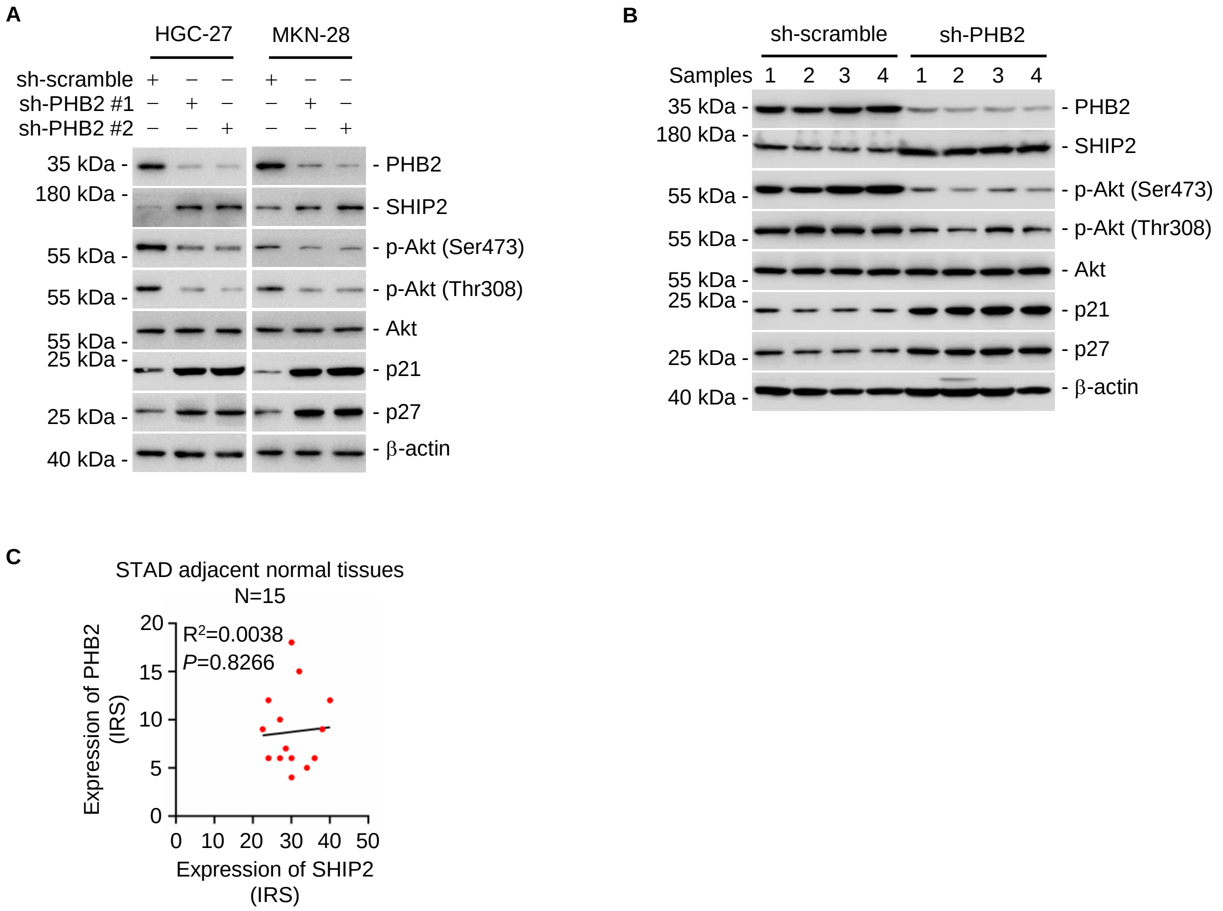

Supplementary Figure 4. PHB2 destabilizes the protein expression of SHIP2, which in turn activates Akt.

A, B, Silencing of PHB2 increased SHIP2 expression and diminished Akt activation in HGC-27 and MKN-28 cells (A) and in MKN-28.sh-PHB2 xenografts (B). Data are representatives of three independent experiments. **C,** PHB2 protein expression was not correlated with SHIP2 protein expression in adjacent normal tissues of GC patient samples (n=15).
